# Antioxidants and Reactive Oxygen Species: Shaping Human Health and Disease Outcomes

**DOI:** 10.3390/ijms26157520

**Published:** 2025-08-04

**Authors:** Charles F. Manful, Eric Fordjour, Dasinaa Subramaniam, Albert A. Sey, Lord Abbey, Raymond Thomas

**Affiliations:** 1 School of Science and the Environment, Grenfell Campus, Memorial University of Newfoundland, Corner Brook, NL A2H 5G4, Canada; 2 Biotron Experimental Climate Change Research Centre, University of Western Ontario, London, ON N6A 5B9, Canada; 3 Department of Plant, Food and Environmental Sciences, Dalhousie University, Truro, NS B2N 5E3, Canada

**Keywords:** inflammation, oxidative stress, nitrosative stress, chronic diseases, oxidation, nanomedicine, precision medicine

## Abstract

Reactive molecules, including oxygen and nitrogen species, serve dual roles in human physiology. While they function as essential signaling molecules under normal physiological conditions, they contribute to cellular dysfunction and damage when produced in excess by normal metabolism or in response to stressors. Oxidative/nitrosative stress is a pathological state, resulting from the overproduction of reactive species exceeding the antioxidant capacity of the body, which is implicated in several chronic human diseases. Antioxidant therapies aimed at restoring redox balance and preventing oxidative/nitrosative stress have demonstrated efficacy in preclinical models. However, their clinical applications have met with inconsistent success owing to efficacy, safety, and bioavailability concerns. This summative review analyzes the role of reactive species in human pathophysiology, the mechanisms of action of antioxidant protection, and the challenges that hinder their translation into effective clinical therapies in order to evaluate potential emerging strategies such as targeted delivery systems, precision medicine, and synergistic therapeutic approaches, among others, to overcome current limitations. By integrating recent advances, this review highlights the value of targeting reactive species in the prevention and management of chronic diseases.

## 1. Introduction

Normal cellular metabolism produces several reactive oxygen and nitrogen species as byproducts. These reactive molecules are critical for cellular redox homeostasis and as signaling molecules in physiological processes, including immune response, gene regulation, and apoptosis. However, when produced in excess or inadequately neutralized by the body’s endogenous antioxidant systems, these species cause oxidative and nitrosative stress, leading to cellular dysfunction and damage Thomas, et al. [1]. This state of cellular redox imbalance, termed oxidative/nitrosative stress, is well known for the incidence of pathologies including cancers, cardiovascular, metabolic, and neurodegenerative diseases. All these pathological conditions share common features of redox imbalance, mitochondrial dysfunction, and chronic inflammation, which are exacerbated by environmental and lifestyle factors that increase cellular reactive species production in humans.

The human body possesses an arsenal of antioxidant systems that work in concert to ensure cellular redox balance [2]. When the body’s endogenous capacity is overwhelmed by the excessive accumulation of reactive species, exogenous antioxidants can provide supplementary protection to prevent oxidative/nitrosative stress and cellular damage. A plethora of preclinical studies demonstrate that exogenous antioxidants can effectively neutralize reactive species, regenerate endogenous enzymes, and restore cellular redox balance, thereby reducing tissue damage and inflammation. Despite promising results, exogenous antioxidant interventions have met with limited success in clinical practice. In other instances, their therapeutic applications have resulted in adverse health effects in subjects [3]. Consequently, in view of the role reactive species play in human health, the development of novel innovative antioxidant therapies to address these shortcomings is the subject of widespread interest.

This review examines the relevance of reactive species in human pathophysiology, the mechanistic basis of antioxidant action, and key limitations impeding the clinical translation of antioxidant therapeutics. It also evaluates the strengths and limitations of current therapeutic approaches aimed at enhancing antioxidant efficacy and safety in preclinical and clinical models, including targeted delivery systems, nanotechnology-based formulations, and synergistic combination therapies. By integrating current research findings, this review provides a clearer comprehension of antioxidant therapeutics to guide the future development of innovative strategies for managing reactive species-driven chronic diseases.

## 2. Bioactive Reactive Species

There are numerous reactive molecules that feature in human pathophysiology [4]. Among these, reactive oxygen species (ROS) specifically refer to highly reactive oxygen-derived molecules, including radicals such as superoxide (O_2_•^−^) and hydroxyl (•OH), among others, and non-radicals such as hydrogen peroxide (H_2_O_2_), singlet oxygen (^1^O_2_), and ozone (O_3_), all of which exist as oxygen-containing molecules (Table 1). Similarly, reactive molecules derived from nitrogen, including radicals such as nitric oxide (•NO) and nitrogen dioxide (•NO_2_), along with non-radicals such as peroxynitrite (ONOO^−^), nitrous acid (HNO_2_), and dinitrogen tetroxide (N_2_O_4_), among others, which contain nitrogen, correspond to reactive nitrogen species (RNS) [5]. The terms oxidative stress and nitrosative stress describe pathological conditions characterized by the overproduction of ROS and RNS, respectively, beyond cellular antioxidant capacity. However, while most RNS also contain oxygen, the reactivity and biological targets of RNS differ from ROS, and the distinction will be made throughout this text, whenever applicable, for simplicity. Additionally, non-free radical reactive molecules generally exhibit lower reactivity compared to their radical counterparts, with the former transformed to free radicals under stress conditions [6]. Table 1 lists the common bioactive reactive molecules, including ROS and RNS, associated with human health.

### 2.1. Production of Reactive Oxygen and Nitrogen Species

Human cells naturally generate ROS and RNS during normal metabolism and immune defense. Additionally, various external stimuli can trigger their production. This section outlines the key sources of bioactive ROS and RNS implicated in human physiology and pathology.

#### 2.1.1. Reactive Oxygen Species (ROS)

##### Endogenous Sources

**Figure 1:**
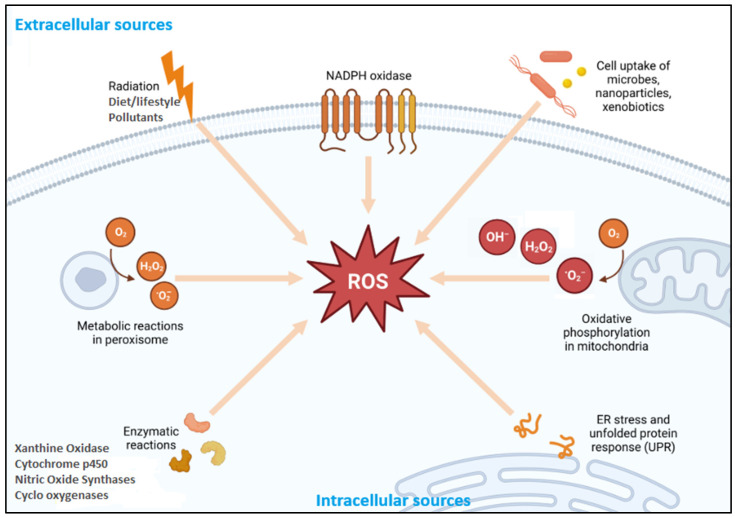
Schematic illustration depicts the various endogenous and exogenous sources contributing to the generation of reactive oxygen species (ROS) within a cell. Key endogenous sources include oxidative phosphorylation in mitochondria; enzymatic reactions involving xanthine oxidase, cytochrome P450, nitric oxide synthases, and cyclooxygenases; and NADPH oxidase activity, metabolic reactions in peroxisomes, and endoplasmic reticulum (ER) stress with unfolded protein response (UPR). Exogenous contributors include radiation, lifestyle and dietary factors, environmental pollutants, and the cellular uptake of microbes, nanoparticles, and xenobiotics. These processes lead to the formation of ROS such as superoxide (O_2_^−^), hydrogen peroxide (H_2_O_2_), and hydroxyl radicals (OH^−^), which can affect cellular homeostasis and contribute to oxidative stress. Modified from [16].

Reactive oxygen species production occurs endogenously via mitochondrial, enzymatic, and non-enzymatic pathways (Figure 1). The mitochondria represent the principal sites of cellular ROS generation during oxidative phosphorylation [7]. Electron leakage at Complexes I and III of the electron transport chain (ETC) furnishes electrons to partially reduce molecular oxygen to O_2_•^−^ in the mitochondria [8]. These species are short-lived, and the enzyme superoxide dismutase (SOD) transforms highly toxic O_2_•^−^ to H_2_O_2_ and O_2_, which are less harmful. Hydrogen peroxide, when untreated by cellular antioxidants, transforms to highly reactive •OH via Fe^2+^-catalyzed Fenton and Haber–Weiss reactions [6]. Mitochondrial ROS production is tightly regulated, balancing its generation and elimination to maintain cellular homeostasis. This regulation involves various factors, including mitochondrial membrane potential, enzymatic systems, and signaling pathways, in balancing signaling roles with the prevention of oxidative damage [9].

Beyond mitochondrial sources, several extra-mitochondrial enzymes are critical contributors to ROS production, particularly in immune and inflammatory responses (Figure 1). Xanthine oxidase, an essential purine metabolic enzyme, generates O_2_•^−^ and H_2_O_2_ during the oxidation of hypoxanthine and xanthine to uric acid [10]. Under ischemia––reperfusion conditions, the conversion of xanthine dehydrogenase to xanthine oxidase amplifies ROS generation, contributing to endothelial injury and tissue damage in cardiovascular and renal pathologies [11]. Similarly, NADPH oxidases (NOX), particularly in phagocytic cells, mediate ROS production during respiratory bursts via NADPH oxidase-catalyzed oxygen reduction [12]. The resulting O_2_•^−^ and derived oxidants play essential roles in microbial killing and inflammatory signaling. Impaired NOX function, as observed in chronic granulomatous disease, leads to recurrent infections, underscoring its physiological importance [13]. Cytochrome P450 enzymes (notably CYP2E1) also contribute to ROS formation via catalytic uncoupling during xenobiotic metabolism, with elevated activity linked to oxidative liver injury in alcohol-associated and drug-induced hepatotoxicity [14]. Furthermore, nitric oxide synthases (NOS), when uncoupled due to deficiencies in cofactors such as tetrahydrobiopterin or substrates like L-arginine, shift from NO• production to O_2_•^−^ generation. This uncoupling exacerbates oxidative stress and is implicated in endothelial dysfunction, hypertension, and atherosclerosis [15]. Together, these enzymatic systems represent spatially distinct but mechanistically interconnected sources of ROS. Their tightly regulated activity is essential for redox signaling and host defense, while their dysregulation leads to multiple oxidative stress-related diseases.

Beyond enzymatic pathways, non-enzymatic and organelle-associated processes significantly contribute to intracellular ROS generation (Figure 1). Peroxisomes produce H_2_O_2_ during fatty acid β-oxidation via flavin-dependent oxidases that transfer electrons to molecular oxygen. Under high metabolic demand or catalase (CAT) insufficiency, excess H_2_O_2_ may accumulate, contributing to oxidative liver injury and peroxisomal disorders [17]. The endoplasmic reticulum (ER) is another ROS source, particularly during oxidative protein folding. Enzymes such as protein disulfide isomerase (PDI) and ER oxidoreductin 1 (Ero1) catalyze disulfide bond formation, transferring electrons to oxygen and generating H_2_O_2_ as a byproduct [18]. Under ER stress, induced by misfolded proteins or calcium dysregulation, the prolonged activation of the unfolded protein response (UPR) enhances ROS production, linking ER dysfunction to metabolic and neurodegenerative diseases [19]. Immune cells, including neutrophils and macrophages, also release ROS, including O_2_•^−^, H_2_O_2_, and HOCl, during respiratory bursts aimed at pathogen destruction [20]. Although essential for host defense, excessive ROS release can damage surrounding tissues, driving chronic inflammation. Under physiological conditions, antioxidant systems maintain redox balance. However, the dysregulation of these systems contributes to oxidative stress and disease pathogenesis.

##### Exogenous Sources

Exogenous factors significantly influence cellular redox balance by either directly generating ROS or enhancing endogenous ROS production through various molecular mechanisms (Figure 1). For instance, ultraviolet (UV) radiation, particularly UVA and UVB, induces the formation of ^1^O_2_, O_2_•^−^, and •OH through the excitation of endogenous chromophores and the initiation of lipid peroxidation cascades [21]. UV exposure also enhances ROS production during the nucleotide excision repair and base excision repair of UV-damaged DNA, contributing to mutagenesis and photoaging [22]. Ionizing radiation, including X-rays, γ-rays, and neutron bursts, interacts with water to generate highly reactive •OH and H_2_O_2_ within cells via radiolysis, resulting in the breakdown of DNA, proteins, and lipids [23].

Environmental pollutants are major external inducers of ROS (Figure 1). Tropospheric ozone, cigarette smoke, and particulate matter (PM2.5) contain or stimulate the formation of free radicals that oxidize cellular components and impair antioxidant defense mechanisms [24,25]. Heavy metals such as cadmium, arsenic, and mercury promote ROS formation by interfering with mitochondrial respiration and depleting endogenous glutathione levels, leading to oxidative damage and inflammation [26]. Additionally, bacterial pathogens and their endotoxins, particularly lipopolysaccharides (LPSs), activate Toll-like receptor 4 (TLR4) and nuclear factor-κB (NF-κB) signaling pathways, upregulating inducible nitric oxide synthase (iNOS) and NOX enzymes and thereby increasing ROS and RNS during the inflammatory response [27].

Dietary and lifestyle factors are increasingly recognized as modifiable contributors to ROS burden (Figure 1). Diets high in oxidized polyunsaturated fats and advanced glycation end-products (AGEs) stimulate ROS through receptor-mediated pathways and mitochondrial overload [28]. Chronic alcohol consumption induces hepatic ROS generation primarily through the upregulation of cytochrome P450 2E1 (CYP2E1), exacerbating oxidative stress and hepatocellular injury [29]. Furthermore, pharmaceutical agents such as non-steroidal anti-inflammatory drugs (NSAIDs), chemotherapeutics (e.g., doxorubicin), and immunosuppressants undergo redox cycling or mitochondrial metabolism, leading to ROS production and antioxidant depletion with prolonged use [30,31].

Collectively, these diverse exogenous stressors contribute to a heightened oxidative load that can overwhelm endogenous antioxidants and drive the pathogenesis of various ROS-related diseases and chronic inflammatory disorders.

#### 2.1.2. Reactive Nitrogen Species (RNS)

##### Endogenous Sources

**Figure 2:**
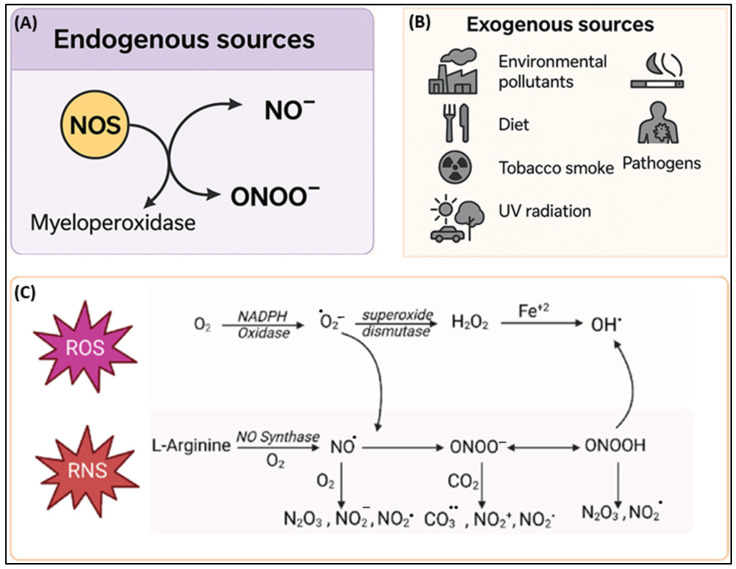
Canonical schematic outlines the sources and biochemical pathways of reactive oxygen species (ROS) and reactive nitrogen species (RNS). (**A**) Endogenous RNS are generated via nitric oxide synthase (NOS), producing nitric oxide (NO^−^) and peroxynitrite (ONOO^−^) through interaction with myeloperoxidase. (**B**) Exogenous sources, such as environmental pollutants, diet, tobacco smoke, pathogens, and UV radiation, also contribute to RNS production. (**C**) The diagram details the interplay between ROS and RNS, highlighting the enzymatic generation of superoxide (O_2_^−^), hydrogen peroxide (H_2_O_2_), hydroxyl radicals (OH^−^), and downstream nitrogen species from L-arginine through NO synthesis and emphasizing their interconnected redox biology. Modified from [36].

The endogenous production of RNS occurs primarily through tightly regulated enzymatic pathways that are crucial for physiological signaling but can contribute to pathological processes under the conditions of oxidative stress (Figure 2). The principal RNS, NO•, is synthesized by NOS enzymes, which catalyze the oxidation of L-arginine to produce NO• and citrulline [32]. There are three isoforms, neuronal NOS (nNOS/NOS1) and endothelial NOS (eNOS/NOS3), that are constitutively expressed and calcium-dependent and mediate critical functions such as neurotransmission and vasodilation, while inducible NOS (iNOS/NOS2) is transcriptionally upregulated as a result of pro-inflammatory cytokines (TNF-α, IL-6, and IL-1β) and microbial products such as lipopolysaccharides (LPSs), generating sustained and high-output NO• during immune responses [33]. Under physiological conditions, NO• promotes vascular homeostasis, inhibits platelet aggregation, and regulates leukocyte adhesion. However, in oxidative environments, NO• combines with O_2_•^−^ to produce ONOO^−^, a potent RNS that nitrates proteins, oxidizes lipids, and damages DNA, thereby contributing to nitrosative stress and tissue injury [34,35].

Other nitrogen species, such as nitrogen dioxide (•NO_2_) along with dinitrogen trioxide (N_2_O_3_), arise from secondary reactions involving NO• and other oxidants [37]. For example, myeloperoxidase, produced by activated neutrophils during inflammation, catalyzes the reaction of nitrite (NO_2_^−^) with H_2_O_2_ to produce •NO_2_, contributing to protein nitration and cellular dysfunction in inflammatory conditions like rheumatoid arthritis and asthma [38,39,40]. Moreover, sources such as xanthine oxidase and the mitochondrial ETC, though primarily known for ROS generation, contribute indirectly to RNS production by supplying O_2_•^−^, which combines with NO• to produce ONOO^−^, thereby linking oxidative and nitrosative stress pathways [41]. The balance between NO• signaling and its conversion into cytotoxic species is a key determinant of redox homeostasis and disease progression.

##### Exogenous Sources

The exogenous sources of RNS arise primarily from environmental exposures and dietary inputs, contributing significantly to systemic nitrosative stress (Figure 2). The inhalation of airborne pollutants, such as cigarette smoke, vehicle exhaust, and industrial emissions, introduces reactive nitrogen oxides (NOₓ), including •NO_2_ and NO•, which readily penetrate lung tissues and initiate inflammatory cascades in the respiratory tract [42]. Ozone (O_3_) and NO_x_ pollutants further interact with endogenous molecules to generate secondary oxidants, compounding respiratory and systemic oxidative stress [43].

Dietary intake is another critical route of exogenous RNS exposure. Processed meats and preserved foods often contain nitrites (NO_2_^−^) and nitrates (NO_3_^−^) as additives. Under the acidic conditions of the stomach, nitrites are reduced to NO• and other nitrogenous intermediates, such as dinitrogen trioxide (N_2_O_3_), which can nitrosate amines to produce genotoxic N-nitroso compounds (NOCs). The presence of heme iron in red meats accelerates this process and has been implicated in colorectal cancer development via lipid and protein nitration [44,45].

Interestingly, nitrate-rich vegetables (e.g., spinach, arugula, beets) also contribute to endogenous RNS generation, but their health impact is modulated by co-occurring antioxidants like vitamin C and polyphenols, which inhibit nitrosation reactions [46]. Pathological states such as inflammation or infection can further amplify both endogenous and exogenous RNS production, tipping redox homeostasis toward nitrosative stress. Understanding these environmental and dietary contributions is vital for developing targeted antioxidant strategies and reducing disease risk linked to nitrosative imbalance.

## 3. Reactive Species in the Pathophysiology of Human Health

Reactive species play dual roles in cellular biology. Under physiological conditions, they serve as critical secondary messengers in redox-regulated signaling pathways, facilitating normal cellular processes. However, when production overwhelms antioxidant defenses, they promote oxidative and nitrosative stress, which leads to cellular dysfunction and disease pathogenesis. Understanding the precise roles and molecular mechanisms by which reactive species contribute to both physiological signaling and pathological damage is essential for elucidating their impact on human health.

### 3.1. ROS in Cellular Signaling and Oxidative Stress

#### 3.1.1. Physiological Roles of ROS

**Figure 3:**
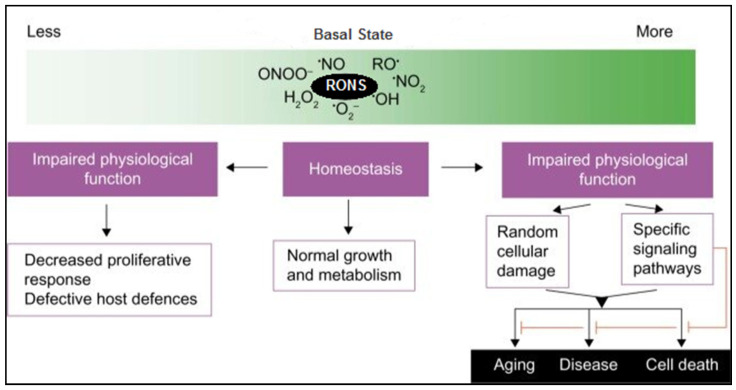
Schematic shows the dual role of reactive oxygen and nitrogen species (RONS) in cellular physiology. At basal levels, RONS support homeostasis, enabling normal growth and metabolism. However, both insufficient and excessive RONS levels lead to impaired physiological function. Low RONS can reduce proliferation and weaken host defenses, while high RONS cause random cellular damage or activate signaling pathways, contributing to aging, disease, and cell death. Modified from [48].

Reactive oxygen species, including O_2_•^−^, H_2_O_2_, and •OH, are traditionally regarded as deleterious byproducts of aerobic metabolism. However, substantial evidence supports their critical function as secondary messengers in redox signaling (Figure 3). At physiological concentrations, ROS regulate diverse intracellular signaling pathways via the reversible oxidation of cysteine residues in key signaling proteins, such as kinases, phosphatases, and transcription factors [47]. These modifications influence multiple cellular processes, including proliferation, differentiation, metabolism, apoptosis, immune responses, and inflammation.

Among various ROS, H_2_O_2_ is particularly important in signal transduction owing to its relative stability, membrane permeability, and selective reactivity [49]. Transient and spatially regulated ROS generation, principally via enzymatic sources such as NADPH oxidases (NOXs), is activated by cytokines, growth factors, and stress. NOX-derived ROS are critical for controlling endothelial function, immune cell activation, and tissue remodeling processes such as angiogenesis [50]. Key redox-sensitive signaling pathways modulated by ROS correspond to the mitogen-activated protein kinase (MAPK) cascade and phosphoinositide 3-kinase (PI3K)/Akt pathway, along with the Janus kinase/signal transducer and activator of the transcription (JAK/STAT) axis [51]. Transcription factors, such as NF-κB, Nrf2, HIF-1α, FOXO proteins, STAT3, and AP-1, orchestrate complex gene expression programs in response to oxidative cues [7,52].

For instance, NF-κB is activated by ROS through the phosphorylation of IκBα by the IκB kinase (IKK) complex, leading to its degradation and the nuclear transference of the NF-κB dimers p65/p50 [53]. This promotes the transcription of pro-inflammatory genes, such as TNF-α, IL-6, and IL-1β; COX-2; iNOS; chemokines; and adhesion molecules to control inflammatory responses [54]. In contrast, Nrf2 functions as a master controller of antioxidant defenses. Under physiological conditions, Nrf2 is sequestered by Kelch-like ECH-associated protein 1 (Keap1) [55]. Upon oxidative stress, ROS oxidize critical cysteine residues in Keap1, releasing Nrf2 to translocate to the nucleus, where it induces the transcription of cytoprotective antioxidant genes, such as NAD(P)H:quinone oxidoreductase 1 (NQO1), heme oxygenase-1 (HO-1), and glutathione S-transferases (GSTs), among others, to protect against oxidative damage [56,57,58].

Other redox-regulated transcription factors contribute to cellular adaptation. AP-1 (composed of c-Jun and c-Fos) regulates genes linked to cell growth and inflammation [59]. STAT3 supports survival, proliferation, and fibrosis in response to redox and inflammatory stress, particularly in the context of chronic disease and cancer [60]. HIF-1α integrates hypoxic and oxidative signals to promote angiogenesis and metabolic reprogramming. FOXO transcription factors are modulated by ROS-dependent post-translational modifications, and control genes modulate cell cycle arrest, apoptosis, and oxidative stress resistance [61,62].

#### 3.1.2. Pathological Roles of ROS

While low-to-moderate ROS levels mediate beneficial signaling effects, excessive ROS production, when antioxidant systems are overwhelmed, results in oxidative stress and cellular injury (Figure 3). Membrane lipids, especially polyunsaturated fatty acids, are vulnerable to ROS-induced peroxidation, generating reactive aldehydes, including 4-hydroxynonenal and malondialdehyde [63]. Although low levels of 4-hydroxynonenal may activate protective Nrf2 responses, higher concentrations denature proteins and nucleic acids, disrupting enzymatic activity and inducing apoptosis [64,65]. Similarly, proteins undergo oxidative modifications including carbonylation, disulfide cross-linking, and methionine or cysteine oxidation, often leading to functional impairment or proteasomal degradation [49]. ROS also attacks DNA, with damage manifesting as strand breaks, abasic sites, and oxidized bases, including 8-oxo-2′-deoxyguanosine, which contributes to mutagenesis and carcinogenesis if not resolved [66].

These findings underscore the duality of ROS as both essential secondary messengers and damaging agents of oxidative stress. Thus, the maintenance of redox homeostasis is essential for cellular integrity since oxidative stress contributes to numerous inflammation- and oxidative stress-related diseases.

### 3.2. RNS in Cellular Signaling and Nitrosative Stress

#### 3.2.1. Physiological Roles of RNS

Reactive nitrogen species, including NO•, ONOO^−^, and •NO_2_, are important mediators of cellular signaling with both physiological and pathological consequences. Like ROS, RNS participate in redox-dependent post-translational modifications that influence signaling pathways and gene expression (Figure 3).

Under physiological conditions, RNS are tightly regulated and play essential roles in multiple systems. Nitric oxide is biosynthesized from L-arginine under the control of the NOS isoforms eNOS, nNOS, and iNOS [32]. In the vasculature, eNOS-derived NO• activates soluble guanylate cyclase, elevating cyclic GMP levels to induce vasodilation, inhibit platelet aggregation, and maintain endothelial integrity and cardiovascular health [67]. In the nervous system, nNOS-derived NO• acts as a neurotransmitter, modulating synaptic proteins and transcription factors through reversible S-nitrosylation, thereby influencing synaptic plasticity, neurovascular coupling, and memory formation [68]. Transcription factors modulated by nNOS-derived NO• include NF-κB, AP-1, and cAMP response element-binding proteins, among others, through direct S-nitrosylation or indirect redox signaling. In the immune system, controlled iNOS activity in macrophages and other immune cells generates NO• as part of the antimicrobial defense, contributing to the clearance of pathogens [69].

RNS also regulate transcription by S-nitrosylating key signaling proteins, such as IKK and AP-1, thereby modulating inflammatory gene expression. Additionally, RNS can modify Keap1, leading to the release and nuclear translocation of Nrf2, and activate antioxidant gene expression. RNS further modulate the action of other transcription factors, including HIF-1α and STAT3, along with p53, integrating redox and nitrosative signals in cellular decision-making [70,71].

#### 3.2.2. Pathological Roles of RNS

When RNS production becomes excessive or dysregulated, as seen in chronic inflammation or oxidative stress, they contribute to nitrosative stress and cellular damage (Figure 3). High levels of NO•, particularly from iNOS, and the formation of ONOO^−^ lead to irreversible modifications of proteins, lipids, and DNA. One significant pathological modification is tyrosine nitration, primarily mediated by ONOO^−^, which alters protein structure and function, disrupts signaling pathways, and impairs enzyme function and cellular homeostasis [72,73]. Tyrosine nitration has been cited in the incidence of cardiovascular, neurodegenerative, and inflammatory diseases [74,75].

Furthermore, while reversible under normal conditions, dysregulated S-nitrosylation can disturb cellular homeostasis and contribute to disease. For instance, the nitrosylation of caspases hinders apoptosis, while modifications of metabolic enzymes impair energy production [76,77]. Persistent nitrosative stress also affects epigenetic regulation by altering DNA methylation and histone structures, further impacting gene expression and cellular function and contributing to genetic mutations and carcinogenesis if left unrepaired [78]. Thus, while RNS are integral to normal cellular physiology, their overproduction disrupts redox-sensitive transcriptional networks and contributes to disease development.

### 3.3. Synergistic Effects of Reactive Oxygen and Nitrogen Species

ROS and RNS frequently act concertedly to exacerbate cellular damage. For instance, the interaction between NO• and O_2_•^−^ not only generates ONOO^−^ but also consumes bioavailable NO•, impairing vasodilation and promoting endothelial dysfunction, a key event in atherosclerosis and hypertension [79]. Furthermore, combined oxidative and nitrosative stress can inhibit DNA repair enzymes such as poly(ADP-ribose) polymerases and 8-oxoguanine glycosylase, perpetuating genomic instability and accelerating disease progression [80].

Table 2 highlights how oxidative, along with nitrosative stress, drives the pathogenesis of various chronic diseases [81]. Their widespread impact underscores the value of targeting redox imbalances in the prevention and treatment of these pathologies. Understanding the spatial and temporal dynamics of ROS and RNS production, as well as their dual physiological and pathological roles, is critical for developing therapeutic strategies to restore redox homeostasis. This includes interventions that enhance endogenous antioxidant systems or pharmacologically scavenge reactive species to mitigate disease-associated damage and restore cellular redox balance. The next section discusses the major antioxidant systems involved in protecting against oxidative and nitrosative damage.

## 4. Antioxidant Modulation of Oxidative and Nitrosative Stress

Oxidative and nitrosative stress perturbs redox signaling and damages vital biomolecules, including lipids, proteins, and nucleic acids. These disruptions significantly contribute to the development of a wide spectrum of human diseases. Antioxidants constitute a broad class of molecules that mitigate these effects by neutralizing reactive species, including ROS and RNS; maintaining redox homeostasis; and preventing macromolecular damage.

### 4.1. Types of Antioxidant Systems

The body’s antioxidant defense network encompasses both endogenous and exogenous systems, each playing complementary roles in cellular protection, signaling modulation, and stress adaptation.

#### 4.1.1. Endogenous Antioxidants

##### Enzymatic Antioxidants

Endogenous enzymatic antioxidants are the primary protection against oxidative and nitrosative stress. The key antioxidants include superoxide dismutase (SOD), catalase (CAT), glutathione peroxidase (GPx), and paraoxonase-2 (PON2) [109]. These enzymes facilitate the detoxification of ROS and RNS through highly regulated catalytic reactions, preserving redox balance and preventing macromolecular damage. For comprehensive discussions of antioxidant systems, readers are directed to these reviews [110,111].

*Superoxide Dismutases*: SODs are metalloenzymes responsible for the dismutation of O_2_•^−^ to H_2_O_2_ and oxygen [112]. Three isoforms exist, corresponding to cytosolic Cu/Zn-SOD (SOD1), mitochondrial Mn-SOD (SOD2), and extracellular SOD (SOD3) [113]. SOD2 is particularly essential in mitochondrial protection, as mitochondria represent primary sites for ROS production. The deficiency or inactivation of SOD2 has been implicated in mitochondrial dysfunction and increased susceptibility to oxidative stress in models of neurodegeneration and cardiomyopathy [114,115].

*Catalase (CAT)*: Catalase, predominantly located in peroxisomes, rapidly decomposes H_2_O_2_ into water and oxygen [116]. Its activity prevents the accumulation of H_2_O_2_, which could otherwise undergo Fenton reactions to produce the highly reactive •OH. High CAT activity is observed in hepatic tissues, highlighting its importance in detoxification processes and hepatocellular protection [108].

*Glutathione Peroxidases*: GPxs constitute a family of selenium-dependent enzymes which reduce lipid hydroperoxides and H_2_O_2_ using reduced glutathione (GSH) as a substrate [117]. GPx1 is ubiquitously expressed and critical in cardiovascular and neuronal antioxidant defense, while GPx4 specifically protects membrane lipids from peroxidation and is essential for embryonic development and ferroptosis suppression [118].

*Paraoxonase-2*: PON2 is an abundant membrane-associated enzyme with lactonase activity, and it is involved in hydrolyzing lipid peroxides and protecting against oxidative stress-induced apoptosis [119]. Interestingly, while PON2 exerts protective effects in oxidative and inflammatory conditions such as atherosclerosis and neuroinflammation, emerging evidence suggests its aberrant overexpression in several malignancies, including glioblastoma, ovarian, and prostate cancers, where it contributes to tumor progression, metabolic reprogramming, and resistance to chemotherapeutics [120,121].

These enzymatic defenses are particularly abundant in organs with high metabolic rates and oxygen needs, including liver, brain, and kidneys, reflecting their vital role in maintaining tissue homeostasis under oxidative pressure.

##### Non-Enzymatic Antioxidants

These antioxidants are free radical scavengers, metal chelators, and redox modulators. They include glutathione, uric acid, bilirubin, and coenzyme Q10 (CoQ10) among others, which either directly neutralize ROS/RNS or regenerate oxidized enzymatic antioxidants, reinforcing cellular antioxidant capacity.

*Glutathione*: GSH is the most abundant intracellular antioxidant [122]. It directly neutralizes ROS, detoxifies electrophilic xenobiotics, and serves as a cofactor for GPxs and glutathione S-transferases [123]. Additionally, GSH contributes to the regeneration of oxidized vitamin C and E, thereby preserving the antioxidant network. GSH depletion is a biomarker of oxidative stress in diseases such as Alzheimer’s disease (AD), Parkinson’s disease (PD), and type 2 diabetes [124,125].

*Uric Acid*: Uric acid functions as a potent scavenger of singlet oxygen and peroxynitrite in the plasma [126]. It represents the final product of purine catabolism. Although high uric acid is linked to gout and cardiovascular risk, at physiological levels, it contributes significantly to the plasma antioxidant capacity, particularly under ischemic or inflammatory conditions [127].

*Bilirubin*: As a heme degradation product, bilirubin has emerged as a cytoprotective antioxidant. It can neutralize ROS and inhibit lipid peroxidation, particularly in neuronal and hepatic tissues [128]. Interestingly, low physiological levels of bilirubin are known to lessen risks of cardiovascular diseases, while mild hyperbilirubinemia, such as in Gilbert’s syndrome, confers protection against oxidative stress-related pathologies [129].

*Coenzyme Q10* (CoQ10): Coenzyme Q10, an oleophilic antioxidant, resides in the inner mitochondrial membrane, where it participates in electron transport and prevents lipid peroxidation [130,131]. It also regenerates vitamin E from its oxidized form and modulates mitochondrial permeability transition. CoQ10 deficiency has been linked to heart failure, neurodegeneration, and aging-related decline, and supplementation has shown promise in reducing oxidative biomarkers in clinical settings [132].

These non-enzymatic antioxidants operate in synergy with enzymatic systems, and their levels are tightly regulated through biosynthesis, recycling, and dietary intake. An imbalance in these components not only heightens susceptibility to oxidative/nitrosative damage but also alters redox-sensitive gene expression profiles that are critical for inflammation, immunity, and apoptosis.

#### 4.1.2. Exogenous Antioxidants

Exogenous antioxidants are derived from dietary sources, and they are essential for maintaining redox homeostasis, particularly when endogenous antioxidant defenses are insufficient, as seen during aging, metabolic stress, environmental toxin exposure, and chronic inflammation [116]. These dietary antioxidants include vitamins, polyphenols, carotenoids, and trace minerals, all of which contribute to oxidative balance either by directly scavenging ROS and RNS or upregulating endogenous antioxidant defense pathways.

##### Vitamins as Antioxidants

*Vitamin C (Ascorbic Acid)*: This is a hydrophilic antioxidant that functions in the aqueous compartments of cells [133]. It directly neutralizes •OH, O_2_•^−^, and peroxyl (ROO•) radicals, and it is involved in regenerating the oxidized forms of other antioxidants, including vitamin E and glutathione [134]. Additionally, it is a cofactor for a number of dioxygenase enzymes pivotal for collagen synthesis, immune function, and epigenetic regulation through histone demethylation. High vitamin C intake is linked to a lowered incidence of cardiovascular disease and cognitive decline, largely due to its capacity to lower endothelial oxidative stress and preserve nitric oxide bioavailability [135].

*Vitamin E (Tocopherols and Tocotrienols)*: This comprises an oleophilic family of antioxidants, having α-tocopherol as the most biologically active form [136]. It is uniquely suited to protect polyunsaturated fatty acids in cellular membranes from peroxidation by intercepting lipid radicals and terminating lipid peroxidation [137]. Vitamin E also modulates redox-sensitive signaling pathways such as protein kinase C, NF-κB, and transforming growth factor-β, contributing to its anti-inflammatory and immunomodulatory functions [138]. Clinical studies report that optimal vitamin E intake reduces the markers of oxidative stress and improves outcomes in pathologies including non-alcoholic fatty liver disease and AD [139].

*Vitamin A and Carotenoids*: Carotenoids, including β-carotene, lycopene, lutein, and zeaxanthin, are lipophilic pigments with potent antioxidant and photoprotective properties [140]. β-carotene serves as a precursor to retinol (vitamin A) and contributes to mucosal immunity and epithelial integrity [141,142]. Lycopene, abundant in tomatoes and watermelon, is particularly effective in quenching ^1^O_2_ and inhibiting lipid peroxidation [143]. Lutein and zeaxanthin are concentrated in the retina and shield ocular tissues from blue light-induced oxidative damage, significantly lowering the incidence of age-related macular degeneration [144,145,146].

##### Polyphenols as Antioxidants

Polyphenols constitute a structurally diverse class of phytochemicals that contain phenol units. They exhibit strong antioxidant and anti-inflammatory effects by regulating redox-sensitive signaling pathways [147,148]. They are subdivided into flavonoids (e.g., quercetin, catechins), phenolic acids (e.g., caffeic acid), stilbenes (e.g., resveratrol), and lignans [149].

For instance, quercetin, found in onions, apples, and tea, has strong radical-scavenging activity and inhibits lipid peroxidation, xanthine oxidase, and NADPH oxidase [150]. It suppresses pro-inflammatory cytokines including TNF-α and IL-6, partly through the control of the NF-κB pathway [151]. Similarly, resveratrol, abundant in red wine and grapes, activates Sirtuin 1 (SIRT1) and Nrf2 pathways, enhances mitochondrial biogenesis, and promotes autophagic flux, all of which confer antioxidant and anti-aging benefits [152]. Epigallocatechin gallate (EGCG) from green tea scavenges ROS, suppresses inflammatory gene expression, and inhibits matrix metalloproteinases implicated in tissue degradation [153]. These polyphenols often exhibit hormetic effects, whereby low-to-moderate doses trigger the transcription of Nrf2 to express endogenous antioxidant responses, contributing to long-term cytoprotection [154].

##### Essential Trace Minerals as Antioxidant Cofactors

Trace minerals, including selenium, zinc, and manganese, play essential roles in maintaining redox homeostasis by supporting catalytic activity and the structural integrity of key antioxidant enzymes. Selenium (Se), a critical cofactor for glutathione peroxidases (GPx) and thioredoxin reductases (TrxR), is vital for peroxide detoxification and redox signaling [155]. Selenium deficiency contributes to reduced immune function and diseases such as Keshan cardiomyopathy, highlighting its physiological importance [156]. Zinc (Zn) contributes structurally to Cu/Zn-superoxide dismutase (SOD1), stabilizes cellular membranes to reduce lipid peroxidation, and modulates redox signaling by downregulating NADPH oxidase activity [157]. Additionally, zinc supports the functioning of DNA repair enzymes, thereby preserving genomic integrity under oxidative stress [158]. Manganese (Mn) is indispensable for the activity of Mn-superoxide dismutase (SOD2), the primary mitochondrial antioxidant enzyme responsible for neutralizing ROS and protecting mitochondrial DNA and proteins. Mn-SOD is especially vital in metabolically active tissues with high oxidative needs, including the brain and heart [159]. Beyond their enzymatic roles, these trace minerals also regulate redox-sensitive transcriptional and translational processes that govern cellular stress responses and adaptation to oxidative environments.

##### Dietary Patterns and Antioxidant Synergy

The health benefits of exogenous antioxidants are best realized in the context of whole dietary patterns rather than isolated supplementation. This is due to synergistic interactions among various nutrients, bioavailability differences, and modulatory effects on gut microbiota and metabolic pathways [160]. The Mediterranean diet contains extra virgin olive oil, fruits, vegetables, legumes, nuts, and moderate red wine, and it has been extensively studied for its antioxidant potential [161]. This diet enhances systemic antioxidant capacity (e.g., increased plasma total antioxidant status, reduced oxidized low-density lipoproteins, lowers C-reactive proteins) and is linked with the reduced incidence of cardiovascular, metabolic, and neurodegenerative diseases [162]. Antioxidant-dense foods, such as berries (anthocyanins), dark chocolate (flavanols), green tea (catechins), turmeric (curcumin), and pomegranate (ellagitannins), have demonstrated the ability to reduce oxidative biomarkers such as malondialdehyde and F_2_-isoprostanes in both observational and interventional studies [163,164]. These dietary elements also support gut redox signaling and microbiota composition, thereby exerting indirect antioxidant effects by regulating microbial metabolites, including short-chain fatty acids and polyphenol derivatives, that regulate innate inflammation and oxidative stress [165].

Taken together, exogenous antioxidants obtained through diet provide critical reinforcement to endogenous redox systems, particularly under pathological or age-associated oxidative stress. A diverse and balanced diet incorporating multiple sources of antioxidant nutrients confers synergistic protection against oxidative and nitrosative damage, thereby contributing to health maintenance, disease prevention, and the promotion of longevity.

### 4.2. Mechanisms of Action of Antioxidants

The generation and detoxification of ROS and RNS by antioxidants are tightly regulated at the transcriptional level through redox-sensitive signaling pathways. Central to this regulation is Nrf2, a transcription factor that modulates cellular redox balance by promoting the secretion of several cytoprotective genes [166]. Once activated, Nrf2 migrates to the nucleus and binds to antioxidant response elements in the promoters of target genes, driving the transcription of key detoxification and antioxidant enzymes, such as GSTs, HO-1, glutamate–cysteine ligase, NQO1, SOD, GPx, and CAT, and TrxR [167,168,169]. Importantly, the activity of Nrf2 is context-dependent. While transient activation confers protection against oxidative damage and inflammation, persistent activation has been associated with tumorigenesis, metastasis, and chemoresistance due to the enhanced cellular survival and suppression of pro-oxidant signaling [170,171]. The dysregulation of Nrf2 signaling is reported to promote chronic diseases, including cancer, neurodegeneration, and metabolic disorders [172], emphasizing the need for the precise modulation of this pathway in antioxidant-based therapies.

In addition to transcriptional control, antioxidants also control gene expression via epigenetic means. These include DNA methylation and histone modifications that influence chromatin structure and gene accessibility [173]. Polyphenols such as curcumin and resveratrol reverse aberrant epigenetic patterns, restoring normal gene activity associated with oxidation and inflammatory response and cell survival [174]. Beyond gene regulation, antioxidants exert protective effects through several interrelated biochemical mechanisms. A primary mechanism involves the direct scavenging of ROS/RNS, thereby neutralizing free radicals and preventing oxidative modifications of cellular macromolecules [175]. Lipid-soluble antioxidants like vitamin E terminate lipid peroxidation chain reactions by transferring hydrogen atoms to lipid peroxyl radicals, thus maintaining membrane integrity, fluidity, and signal transduction [176,177]. Similarly, water-soluble antioxidants, including vitamin C, neutralize hydroxyl and peroxyl radicals and regenerate reduced antioxidants to their active forms.

Another essential mechanism involves antioxidant recycling, which enhances the efficiency of the redox defense system. Vitamin C regenerates oxidized vitamin E, while coenzyme Q restores other lipid-phase antioxidants, forming a cooperative network that sustains antioxidant capacity under stress conditions [178,179]. This synergy highlights the interdependence of antioxidant systems in maintaining cellular redox balance. Antioxidants also bolster endogenous enzymatic antioxidant defenses, including SOD, GPx, and CAT. These enzymes promote the detoxification of O_2_•^−^ and H_2_O_2_ to less reactive species. Their activity depends on essential cofactors such as selenium (for GPx) and manganese or copper/zinc (for SOD) [180]. Notably, certain dietary antioxidants such as flavonoids and phenolic acids can upregulate the gene expression or activity of these enzymes, thereby reinforcing intrinsic defense mechanisms [181,182].

In parallel, many antioxidants exhibit anti-inflammatory properties by modulating redox-sensitive signaling pathways. For instance, ROS generated during immune responses can activate NF-κB, which regulates the pro-inflammatory cytokines (TNF-α, IL-1, and IL-6) and adhesion molecules. Polyphenolic antioxidants such as EGCG and curcumin inhibit NF-κB activation, thereby attenuating inflammatory cascades and offering therapeutic potential in chronic inflammatory diseases [50,182].

Table 3 summarizes key endogenous and exogenous antioxidants, their representative examples, sites of action, and mechanisms of antioxidant activity. Collectively, the combined actions of transcriptional regulation, enzymatic modulation, radical scavenging, and inflammatory suppression constitute a multifaceted antioxidant defense system. The dynamic interplay between endogenous and exogenous antioxidants provides a comprehensive framework for maintaining redox homeostasis and mitigating oxidative and nitrosative stress-related pathologies. Elucidating these pathways is fundamental to advancing targeted antioxidant strategies in clinical settings.

## 5. Application of Antioxidant-Based Therapies

Building on their mechanistic versatility, antioxidant-based therapies have been widely explored for their potential in mitigating oxidative and nitrosative stress in human pathologies. These include cardiovascular and neurodegenerative disorders, cancer, diabetes, inflammatory conditions, ocular and skin diseases, reproductive dysfunction, and impaired physical performance [81,183]. By neutralizing excessive reactive oxygen and nitrogen species, antioxidants help preserve cellular integrity, attenuate inflammation, and restore redox homeostasis, thereby contributing to disease prevention and symptom alleviation.

For instance, in skin health and antiaging, the aging process is accelerated by oxidative stress, which causes cumulative cellular and tissue damage. Research evidence demonstrates that antioxidants can extend lifespan in model organisms; for example, compounds such as spermidine and resveratrol stimulate autophagy, thereby protecting cells from oxidative injury [184]. In dermatology, both endogenous and exogenous antioxidants play critical roles in counteracting aging skin and environmental damage, particularly damage caused by UV radiation. UV exposure significantly increases the production of ROS, which accelerates skin aging, wrinkle formation, and photoaging. The skin’s intrinsic antioxidant defense system, including enzymatic antioxidants such as SOD, CAT, GPx, and PON2, works to neutralize ROS and maintain redox homeostasis. Notably, PON2 expression is relatively low in benign actinic keratosis but markedly elevated in squamous cell carcinoma, highlighting its complex role in skin oxidative stress and carcinogenesis linked to aging and UV exposure [185]. Alongside these endogenous systems, exogenous antioxidants such as vitamins C and E, coenzyme Q10, and polyphenols from green tea, berries, and other plant extracts help scavenge ROS and reduce UV-induced skin damage [186,187]. Strengthening both intrinsic and extrinsic antioxidant defenses remains a promising approach to support skin health and mitigate visible signs of aging. Table 4 provides a detailed overview of therapeutic applications, mechanisms, and relevant antioxidant compounds across several disease domains.

While preclinical studies consistently highlight protective benefits of antioxidants in various models of disease, translation of these benefits into clinical practice has yielded mixed outcomes [305]. This discrepancy underscores the need to critically examine the limitations and controversies surrounding antioxidant therapy. Despite promising experimental data, human trials have produced inconsistent results, raising concerns about clinical efficacy, optimal dosing strategies, pharmacokinetics, safety, and the inherent biological complexity of antioxidant interventions as outlined below. Additionally, the dualistic role of ROS and RNS, as both harmful byproducts and essential signaling molecules, complicates the rationale for broad-spectrum antioxidant use [306].

### 5.1. Efficacy and Safety Concerns

One of the most persistent hurdles in the field of antioxidant therapy is the difficulty in achieving both effective and safe outcomes [306]. Several antioxidants, including vitamin C, vitamin E, and polyphenols, face significant pharmacokinetic barriers that limit their clinical utility. For example, vitamin C absorption is saturable, and any excess is rapidly excreted, making it difficult to sustain therapeutic plasma concentrations [307,308]. Similarly, polyphenols, despite their abundance in fruits and teas, are extensively metabolizes in the liver and gut, resulting in low systemic bioavailability along with limited tissue penetration [309,310].

Concerns about safety are equally pressing. High-dose supplementation of certain antioxidants can lead to unintended and sometimes serious adverse effects. Vitamin E, for instance, is linked to heightened incidence of hemorrhagic stroke and all-cause mortality when consumed in excessive amounts [311]. Selenium, another widely used antioxidant, can be toxic at high doses, causing selenosis, a condition characterized by gastrointestinal disturbances, hair loss, and neurological symptoms [312]. Such adverse outcomes have been observed in large-scale clinical studies, including the SELECT trial, which reported increased risks of prostate cancer and diabetes with high-dose vitamin E and selenium supplementation (NCT00006392).

Individual variability further complicates the picture. Genetic differences in antioxidant enzyme systems, pre-existing health conditions, and interactions with other medications all influence how patients respond to antioxidant therapy. This diversity underscores the necessity for personalized dosing and vigilant monitoring instead of a universal one-size-fits-all approach [313].

### 5.2. Inconsistent Clinical Trial Outcomes

The translation of antioxidant therapy from promising epidemiological and preclinical findings to successful clinical outcomes has proven elusive. While observational studies and laboratory experiments propose that diets containing antioxidants decrease the incidence of chronic diseases, large-scale randomized clinical trials have largely failed to confirm these benefits when antioxidants are administered as supplements [306]. For example, early studies indicated that high intake of vitamins C and E might protect against atherosclerotic cardiovascular disease, yet subsequent trials involving thousands of participants did not demonstrate a reduction in cardiovascular events or mortality with supplementation [314,315,316].

Several factors help explain these inconsistencies. There is a fundamental difference between consuming antioxidants as part of whole foods and as isolated supplements. The food matrix, which includes fiber, other micronutrients, and non-antioxidant bioactives, may produce synergistic effects that are not replicated by single-agent supplementation [317]. Furthermore, the timing, dosage, and duration of antioxidant therapy are important factors. High-dose supplementation over short periods may not reverse decades of accumulated oxidative damage, and in some cases, such as β-carotene in smokers, supplementation has actually heightened the incidence of lung cancer [318]. These reports highlight the situational and sometimes adverse effects of antioxidants, challenging the notion of a universal benefit.

Additionally, many clinical trials may not have been of sufficient duration to detect meaningful benefits, particularly for chronic diseases that develop over many years. The choice of antioxidant compounds in trials has often been dictated by availability rather than demonstrated efficacy, further complicating the interpretation of results.

### 5.3. Controversies in Cancer Therapy

The administration of antioxidants as adjuvants during cancer therapy remains in the field [319]. Chemotherapy and radiation therapy impart their cytotoxic effects, in part, by generating ROS that damage tumor cell DNA. The rationale for antioxidant supplementation is to protect normal tissues from collateral oxidative injury, thereby reducing side effects and improving patient well-being. However, this protective effect is not selective; antioxidants may also shield malignant cells from therapy-induced oxidative damage, potentially diminishing the effectiveness of cancer treatments [320].

Systematic reviews and meta-analyses have brought this dilemma into sharp relief. Some studies suggest that antioxidants, including vitamin A, β-carotene, and CoQ10, reduce treatment toxicity and improve patient outcomes [321,322], yet others find no benefit or even report harm [323,324]. For instance, vitamin A supplementation has been linked to higher risks of death and recurrence in some cancer cohorts, while β-carotene supplementation exacerbated lung cancer and mortality incidence among smokers [325]. A meta-analysis found that antioxidant use during chemotherapy and radiation was associated with increased mortality rates and reduced chances of cancer-free survival among breast cancer survivors. These conflicting results have led many oncologists to advise against the administration of antioxidant supplements during active cancer treatment until more definitive evidence is available [319].

### 5.4. Pro-Oxidant Effects and Redox Imbalance

A paradoxical and often overlooked challenge in antioxidant therapy is the potential for certain antioxidants to act as pro-oxidants under specific circumstances. The redox environment within cells is delicately balanced, and excessive supplementation can disrupt this equilibrium. High doses of vitamin C and E, for example, may not only fail to quench reactive oxygen species but may actually promote oxidative stress by interfering with physiological redox signaling or by participating in redox cycling reactions [306,314,326]. This is important in the context of exercise, where ROS serve as essential signaling molecules that mediate beneficial adaptations such as mitochondrial biogenesis. Suppressing these signals with antioxidant supplements can impair the body’s adaptive response to exercise [327,328].

Moreover, endogenous antioxidant enzymes, including SOD and CAT, are far more efficient and specific than exogenous small-molecule antioxidants. This raises important questions about the ability of dietary supplements to meaningfully influence redox status in vivo, especially in individuals with robust endogenous defenses [306,329].

### 5.5. Biological Complexity and Limitations in Trial Design

The biological landscape of oxidative stress is characterized by intricate feedback loops, redundancy, and compartmentalization. Diseases such as hypertension, neurodegeneration, and cancer involve multiple sources of ROS and diverse cellular targets, making it unlikely that a single antioxidant could provide universal benefit. Patient heterogeneity, including genetic background, baseline antioxidant status, and the occurrence of comorbidities, further complicates the interpretation of trial results [306,330,331].

Another major limitation is the lack of reliable, disease-specific biomarkers of oxidative stress. Commonly used markers, including malondialdehyde or F2-isoprostanes, offer only a crude estimate of systemic oxidative burden and may not reflect tissue-specific pathology or the dynamic nature of redox changes [332]. This makes it challenging to select appropriate patient populations, monitor therapeutic responses, and tailor interventions effectively.

Trial design itself poses additional challenges. Many studies have not adequately accounted for confounding factors such as dietary patterns, lifestyle, or genetic predispositions. Moreover, the endpoints chosen in trials may not be sensitive enough to detect subtle but clinically meaningful changes in oxidative stress or disease progression [306].

Persistent gaps between antioxidant mechanisms and clinical outcomes reflect deeper complexities in redox biology and disease pathology. Addressing these challenges requires a shift in how antioxidant interventions are conceptualized and applied within therapeutic contexts. The next section explores emerging strategies designed to overcome these barriers and enhance the clinical utility of antioxidant-based therapies.

## 6. Emerging Advances in Antioxidant Research: Innovative Solutions to Clinical Limitations

Despite extensive evidence demonstrating the therapeutic potential of antioxidants across a wide range of diseases, significant limitations continue to hinder their clinical efficacy. Challenges such as poor bioavailability, rapid metabolic degradation, lack of target specificity, and unpredictable redox behavior have contributed to inconsistent outcomes in randomized controlled trials and meta-analyses. In response to these issues, a variety of innovative approaches have emerged in recent years to optimize antioxidant delivery, enhance therapeutic specificity, and individualize treatment strategies [333]. These advances represent a paradigm shift in antioxidant therapy and are described below in detail.

### 6.1. Nanotechnology-Driven Antioxidant Delivery

Nanotechnology has significantly advanced the delivery of antioxidant compounds by addressing key pharmacokinetic and pharmacodynamic barriers. Nano-formulations, including nanoparticles, liposomes, dendrimers, and solid lipid carriers, offer protection against premature degradation, increase aqueous solubility, and enable targeted or sustained release at specific tissue sites [334]. For a comprehensive review of the application of nanotechnology in antioxidant therapies, the reader is directed to the following reviews: [335,336].

For example, curcumin-loaded polymeric nanoparticles have demonstrated superior bioavailability along with neuroprotective efficacy in animal models of AD by reducing β-amyloid aggregation and improving cognitive outcomes [337,338]. Similarly, clinical studies confirmed the benefits of nanocarriers to enhance the pharmacological profile of natural antioxidants. A recent randomized controlled trial involving type 2 diabetic patients showed that nano-encapsulated resveratrol and curcumin significantly improved insulin sensitivity and reduced oxidative biomarkers compared to their free forms [339]. In oncology, tocotrienol-loaded nanocarriers have selectively targeted tumor cells and induced apoptosis without affecting normal tissue in preclinical breast and pancreatic cancer models [340,341]. Meta-analyses also support the efficacy of nanotechnology-based antioxidant delivery in improving tissue uptake and therapeutic outcomes across cardiovascular and neurological diseases [342].

### 6.2. Synergistic and Combination Therapies

The complexity of oxidative stress-related pathologies often requires therapeutic interventions that extend beyond monotherapy. Consequently, synergistic regimens combining antioxidants with pharmaceuticals, immunomodulators, or nutraceuticals have gained considerable attention [343]. These combination approaches not only enhance efficacy through complementary mechanisms but also mimic the endogenous antioxidant network. For instance, a meta-analysis encompassing 19 randomized controlled trials demonstrated that combining antioxidants such as vitamin C, vitamin E, and Se, along with N-acetylcysteine, with standard chemotherapeutics reduced treatment-induced toxicity while preserving anticancer efficacy [344]. In inflammatory diseases such as rheumatoid arthritis, co-administration of vitamin E along with omega-3 fatty acids resulted in greater reductions in pro-inflammatory cytokines and oxidative stress markers than either compound alone [345]. Similarly, EGCG, a green tea polyphenol, synergistically enhances the efficacy of NSAIDs in experimental models of inflammatory bowel disease by reducing mucosal damage and suppressing oxidative and nitrosative stress [346,347]. These reports emphasize the value of combination therapies to target multiple disease pathways and achieve superior clinical outcomes.

### 6.3. Personalized and Precision Antioxidant Medicine

The inter-individual variability in redox homeostasis, disease etiology, and response to antioxidant supplementation has stimulated interest in personalized medicine approaches. Advances in genomics, metabolomics, and microbiome science now enable more refined and targeted antioxidant interventions. Genetic polymorphisms in antioxidant enzymes, including superoxide dismutase 2 (SOD2 Val16Ala) and glutathione peroxidase 1 (GPX1), are known to modulate susceptibility to oxidative damage and influence disease outcomes [348,349].

Emerging clinical studies are using genetic screening to tailor antioxidant therapy. For example, individuals harboring the SOD2 Ala/Ala genotype have shown enhanced responses to mitochondria-targeted antioxidants such as MitoQ, with improved endothelial function and reduced vascular oxidative stress [350]. Moreover, patient-specific interventions based on metabolomic and microbiome profiles have demonstrated efficacy in metabolic syndrome. Targeted supplementation with ellagitannin-rich polyphenols led to improved insulin sensitivity and lipid profiles in a metabolically stratified cohort [351,352]. Precision dosing algorithms that account for age, sex, comorbidities, genetic variants, and metabolic status are currently under development to further refine antioxidant pharmacotherapy. Ongoing clinical trials are evaluating redox-based tumor signatures as predictive biomarkers for tailored antioxidant co-administration in cancer therapy (e.g., NCT03529110) [353,354].

### 6.4. Enzyme-Mimetic Antioxidants

To overcome the stoichiometric limitations of classical antioxidants, researchers have developed small-molecule mimetics of endogenous antioxidant enzymes, including SOD, CAT, and GPx [306]. These mimetics offer catalytic activity, target specificity, and sustained action.

One notable example is GC4419 (avasopasem manganese), a SOD mimetic that significantly reduced the severity and duration of oral mucositis in subjects receiving radiotherapy for head and neck cancer in recent phase III clinical trials [355,356]. Another example is Ebselen, a selenium-containing glutathione peroxidase mimic, which has demonstrated neuroprotective effects in clinical trials targeting bipolar disorder and noise-induced hearing loss [357,358,359]. Preclinical models of stroke, myocardial infarction, and radiation injury have also shown that these compounds provide broad-spectrum cytoprotection by restoring redox homeostasis and modulating pro-inflammatory pathways [360].

### 6.5. Redox Biomarkers and Real-Time Monitoring Tools

A key advancement in improving antioxidant therapy lies in the ability to assess oxidative stress levels in real time [361,362]. The integration of redox biomarkers into clinical and translational research has facilitated risk stratification, therapeutic monitoring, and early detection of treatment failure. Biomarkers including 8-hydroxy-2′-deoxyguanosine, F2-isoprostanes, malondialdehyde, and glutathione disulfide-to-glutathione ratios are being increasingly utilized in clinical trials as surrogate endpoints of oxidative stress [363,364]. Moreover, emerging technologies such as biosensors and wearable diagnostic platforms are being designed to provide continuous, non-invasive measurements of redox biomarkers. These innovations hold promise in enabling dynamic dose adjustment and timely intervention, ultimately enhancing the efficacy and safety of antioxidant-based therapies [365].

### 6.6. Future Outlook

Collectively, these innovations signify a critical turning point in antioxidant therapeutics. The integration of nanotechnology, synergistic treatment modalities, personalized medicine, enzyme-mimetic compounds, and real-time biomonitoring addresses many of the longstanding challenges that have impeded clinical translation. These approaches not only improve antioxidant pharmacokinetics and pharmacodynamics but also align therapeutic strategies with individual patient biology. With progress in understanding the complexity of redox biology and the development of advanced delivery and monitoring systems, antioxidant therapy is poised to evolve from a generalized supplement-based model into a targeted, evidence-based, and clinically actionable paradigm. Future studies incorporating multi-omics technologies, digital health tools, and large-scale longitudinal trials will be essential in validating these advances and translating them into routine clinical practice.

## 7. Conclusions

Oxidative and nitrosative stress features predominantly in pathogenesis of numerous chronic diseases, underscoring the therapeutic potential of antioxidant-based strategies. However, clinical outcomes have been inconsistent, reflecting challenges related to bioavailability, individual variability, and the complex dual roles of reactive species in human pathophysiology. Looking forward, the future of antioxidant therapy lies in an integrated approach: discovery of novel bioactive compounds with precise redox-modulating activity, advancement of nanotechnology-based delivery platforms to enhance tissue targeting and stability, and adoption of precision medicine to tailor interventions to individual genetic and metabolic profiles. These innovations offer a promising path toward safer, more effective, and personalized therapies for managing oxidative stress-related diseases.

## Figures and Tables

**Table 1:** Common bioactive reactive species associated with human health and diseases.

Radicals	Formula	Non-Radicals	Formula
Superoxide anion *	O_2_•^−^	Hydrogen peroxide *	H_2_O_2_
Hydroxyl radical *	•OH	Singlet oxygen *	^1^O_2_
Nitric oxide ^†^	•NO	Ozone/trioxygen *	O_3_
Nitrogen dioxide ^†^	•NO_2_	Organic hydroperoxides *	ROOH
Organic radicals	R•	Hypochlorite *	ClO^−^
Peroxyl radicals *	ROO•	Hypochlorous acid *	HOCl
Alkoxyl radicals *	RO•	Peroxynitrite ^†^	ONOO^−^
Thiyl radicals	RS•	Akyl peroxynitrite ^†^	ROONO
Sulfonyl radicals	ROS•	Nitrosoperoxycarbonate anion ^†^	O=NOOCO_2_^−^
Thiyl peroxyl radicals *	RSOO•	Nitrocarbonate anion ^†^	O_2_NOCO_2_^−^
Hydroperoxyl *	HOO•	Dinitrogen dioxide ^†^	N_2_O_2_
Nitrogen dioxide ^†^	•NOO	Nitronium ^†^	NO_2_^+^
		Dinitrogen trioxide ^†^	N_2_O_3_
		Nitrous acid ^†^	HNO_2_
		Nitroxyl anion ^†^	NO^−^
		Nitrosyl cation ^†^	NO^+^
		Nitryl chloride ^†^	NOCl
		Dinitrogen tetraoxide ^†^	N_2_O_4_
		Disulfides	RSSR

* Reactive oxygen species; ^†^ reactive nitrogen species.

**Table 2:** Chronic human diseases associated with persistent oxidative stress and nitrosative stress.

Disease Category	Specific Diseases	Role of Oxidative/Nitrosative Stress	Study Limitations	References
Cardiovascular Diseases	Atherosclerosis, Hypertension, Heart Failure	Oxidative stress induces LDL oxidation, leading to foam cell formation and plaque buildup. ROS/RNS impair nitric oxide (NO) signaling, causing endothelial dysfunction and vascular tone dysregulation. In heart failure, oxidative/nitrosative stress promotes myocardial remodeling and apoptosis.	Predominantly based on animal or in vitro models; causal pathways in humans remain underexplored.	[82,83,84]
Neurodegenerative Diseases	Alzheimer’s Disease, Parkinson’s Disease, Huntington’s Disease	ROS and RNS contribute to neurotoxicity by inducing lipid peroxidation, protein aggregation (e.g., amyloid-beta, alpha-synuclein), and mitochondrial dysfunction. These species activate microglia, leading to chronic neuroinflammation and synaptic loss.	Translational challenges from animal models to humans; complexity of disease progression limits mechanistic clarity.	[85,86,87,88]
Cancers	Lung, Breast, Colon, Prostate, etc.	Oxidative and nitrosative stress contribute to tumor initiation by inducing DNA mutations and genomic instability. They also activate pro-oncogenic transcription factors (e.g., NF-κB, AP-1) and promote angiogenesis and tumor progression.	Multifactorial nature of cancer makes it difficult to attribute causality to oxidative stress alone.	[89]
Diabetes	Type 1 and Type 2 Diabetes	In type 1 diabetes, ROS mediate autoimmune destruction of pancreatic beta cells. In type 2 diabetes, oxidative stress impairs insulin signaling, enhances adipose tissue inflammation, and contributes to endothelial dysfunction.	Limited longitudinal human studies; antioxidant therapy trials yield inconsistent results.	[90,91]
Chronic Inflammatory Diseases	Rheumatoid Arthritis, Inflammatory Bowel Disease (IBD)	ROS and RNS sustain chronic inflammation by activating NF-κB and MAPK pathways, leading to cytokine overproduction and tissue damage in joints or the gastrointestinal tract. They also perpetuate immune dysregulation.	Confounding from co-existing inflammatory processes; lack of robust antioxidant-targeted clinical trials.	[92,93,94]
Respiratory Diseases	COPD, Asthma	Inhaled pollutants and allergens enhance ROS/RNS generation, which triggers epithelial injury, mucus hypersecretion, airway remodeling, and inflammation. NO-derived species exacerbate bronchoconstriction and hyperreactivity.	Evidence largely from animal models and ex vivo studies; need for large-scale human trials.	[95,96,97]
Renal Diseases	Chronic Kidney Disease (CKD), Acute Kidney Injury (AKI)	ROS and RNS mediate tubular cell apoptosis, glomerular injury, and interstitial fibrosis. They impair mitochondrial function and activate profibrotic signaling pathways (e.g., TGF-β).	Lack of standardization in oxidative stress biomarkers; heterogeneous patient populations in studies.	[98,99]
Age-Related Diseases	Macular Degeneration, Sarcopenia	Accumulated oxidative damage to retinal pigment epithelium (in AMD) and skeletal muscle proteins (in sarcopenia) disrupts tissue integrity and accelerates functional decline with age.	Multifactorial influences of aging (e.g., metabolic, genetic) complicate interpretation of oxidative stress roles.	[100,101]
Liver Diseases	Non-alcoholic liver disease (NAFLD), Cirrhosis, Hepatitis	Hepatic steatosis and inflammation are driven by lipid peroxidation and mitochondrial ROS/RNS production. Oxidative stress triggers stellate cell activation, leading to fibrosis and cirrhosis.	Few well-powered clinical trials evaluating antioxidant interventions; heterogeneity in liver disease subtypes.	[102]
Muscle Diseases	Muscular Dystrophy, Sarcopenia	ROS and RNS disrupt calcium homeostasis, impair mitochondrial respiration, and induce muscle fiber necrosis. Chronic oxidative stress leads to muscle wasting and fatigue.	Studies limited by small sample sizes and short durations; genetic variability complicates interpretation.	[103,104]
Infectious Diseases	HIV, Sepsis	Overproduction of ROS/RNS during infection leads to oxidative damage of immune and endothelial cells, contributing to immunosuppression and multiorgan failure. Nitrosative stress disrupts mitochondrial and barrier function.	Difficult to isolate oxidative stress from pathogen-induced effects; clinical data remain inconclusive.	[105,106]
Autoimmune Diseases	Systemic Lupus Erythematosus (SLE), Multiple Sclerosis	ROS and RNS act as secondary messengers that enhance autoreactive immune responses, promote DNA fragmentation, and damage target organs such as the kidneys (SLE) or CNS (MS).	Mechanistic overlap between immune activation and oxidative stress; lack of validated redox biomarkers.	[104,107,108]

ROS = Reactive oxygen species; RNS = reactive nitrogen species; NF-κB = nuclear factor kappa-light-chain-enhancer of activated B cells; TGF-β = transforming growth factor beta; AP-1 = activator protein-1; MAPK = mitogen-activated protein kinase; AMD = age-related macular degeneration; CNS = central nervous system; MS = multiple sclerosis; SLE = systemic lupus erythematosus.

**Table 3:** Summary of major endogenous and exogenous antioxidants and their mechanisms of action.

Antioxidant Class	Representative Examples	Site of Action	Primary Mechanisms of Action
Endogenous Antioxidants			
Enzymatic	Superoxide Dismutase (SOD1, SOD2, SOD3)	Cytosol (SOD1), mitochondria (SOD2), extracellular space (SOD3)	Converts superoxide (O_2_•^−^) to hydrogen peroxide (H_2_O_2_)
	Catalase (CAT)	Peroxisomes	Decomposes H_2_O_2_ into water and oxygen
	Glutathione Peroxidase (GPx)	Cytosol and mitochondria	Reduces H_2_O_2_ and lipid hydroperoxides using glutathione (GSH)
	Paraoxonase-2 (PON2)	Mitochondrial and plasma membranes	Inhibits lipid peroxidation and modulates redox signaling
Non-enzymatic	Glutathione (GSH)	Cytosol, nucleus, mitochondria	Reduces peroxides and regenerates oxidized vitamins C and E
	Uric Acid	Plasma, extracellular fluid	Scavenges singlet oxygen and peroxynitrite; protects against lipid and DNA oxidation
	Bilirubin	Plasma, liver, brain	Scavenges peroxyl radicals; anti-inflammatory and neuroprotective functions
	Coenzyme Q10 (Ubiquinone)	Mitochondrial membranes	Prevents lipid peroxidation in mitochondrial inner membrane; regenerates vitamin E
Exogenous Antioxidants			
Vitamins	Vit C (Ascorbic acid)	Cytosol, plasma	Neutralizes ROS (•OH, ROO•); regenerates vitamin E and GSH
	Vit E (α-Tocopherol)	Lipid membranes	Terminates lipid peroxidation chain reactions; stabilizes membrane integrity
	Vit A	Eyes, skin, adipose tissue	Quench singlet oxygen; protect against UV- and light-induced oxidative damage
Polyphenols	Quercetin, Resveratrol, EGCG	Cytosol, nucleus	Scavenge ROS/RNS; inhibit pro-inflammatory enzymes; activate Nrf2 signaling
Carotenoids	β-Carotene, Lutein, Zeaxanthin, Lycopene	Retina, skin	Absorb blue light; quench singlet oxygen and prevent lipid oxidation
Trace Minerals	Selenium	All tissues (especially liver and kidney)	Cofactor for GPx and thioredoxin reductase; supports peroxide detoxification
	Zinc	Cytosol, membranes	Cofactor for Cu/Zn-SOD; stabilizes proteins and membranes; inhibits NADPH oxidase
	Manganese	Mitochondria	Cofactor for Mn-SOD; prevents mitochondrial oxidative damage

Nrf2 = Nuclear factor erythroid 2-related factor 2; EGCG = epigallocatechin gallate.

**Table 4:** Therapeutic applications of antioxidants in human disease management: key outcomes and limitations.

**Condition**	Antioxidant	Mechanism	Disease	Preclinical Outcomes	Clinical Outcomes	Inconsistent/Limited Outcome
Cardiovascular System	Vit C, Vit E, Flavonoids, Resveratrol	Inhibit LDL oxidation, boost NO•, reduce vascular inflammation	Atherosclerosis, hypertension, endothelial dysfunction	Resveratrol reduced atherosclerosis induced by HFD in mice [188] Resveratrol reduced oxidative stress in mice submitted to atherogenic diet leading to CV protection [189]. Meta-analysis: Flavonols reduced atherosclerotic lesion in apolipoprotein E-deficient mice [190]. Vit C suppressed vascular endothelial cell damage; improved endothelial function in adult septic mice [191].	Vit C reduced blood pressure in short-term RCT [192]. Meta-analysis: Improved endothelial function and cardiovascular health with resveratrol [193]. RCT: Antioxidant polyphenol [194] and flavan-3-ol-rich food [195] supplementation reduced oxidative stress and improved CV health.	Meta-analyses: No evidence in support of vitamin and antioxidant supplements for prevention of CVDs [196,197,198]. Meta-analysis: High-dose Vit E supplementation linked to hemorrhagic stroke [199].
Neurodegeneration	Curcumin, CoQ10, Resveratrol, Melatonin, Vit E	Scavenge ROS, stabilize mitochondria, reduce protein aggregation	AD, PD, HD	Curcumin reduced and reversed existing amyloid pathology and associated neurotoxicity in mouse model of AD [200]. CoQ10 suppressed progression of neuro-degeneration and restored synaptic plasticity and mitochondrial function in aged β-amyloid (Aβ)-induced AD rats [201,202,203].	CoQ10 slowed PD progression in early-stage RCT [204,205]. RCT: Ubiquinol-10 (reduced form of CoQ10) conferred motor benefit in early PD [206].	Meta-analysis: No cognitive benefit or prevention of dementia in AD with Vit E supplementation due to low CNS bioavailability [207,208].
Cancer Prevention	EGCG, Curcumin, Sulforaphane, Selenium	Support detox enzymes, induce apoptosis, inhibit angiogenesis	Breast, prostate, colon, lung	EGCG suppressed tumor growth and prostate cancer in xenografts [209,210]. Meta-analyses: Curcumin inhibited prostate cancer growth in animal models [211,212].	RCT: Curcumin effective for treatment of oral leukoplakia [213]. RCT: Curcumin effective for treating oral leukoplakia [214].	RCT (SELECT): Increased prostate cancer risk with selenium and vitamin E [215]. Meta-analysis: Curcumin had no significant/positive effect on the therapy of malignant diseases due to limited bioavailability [216,217].
Skin Health	Vit C, Vit E, Astaxanthin (AST), CoQ10, Polyphenols	Scavenge UV-ROS, enhance collagen, reduce matrix metalloproteinases (MMP) activity	Photoaging, wrinkles, hyperpigmentation	AST reduced/ prevented UV-induced photoaging and wrinkles in skin of hairless mice [218,219] Vit C exerted anti-aging properties on human fibroblast cells [220]. Vit C improved dermal collagen in hairless skin mice [221].	Topical Vit C improved photodamaged skin and ultrastructure in RCT [222]. Topical application of Vit C in RCT significantly increased the density of dermal papillae in aged human skin [223].	Inconsistent outcomes in meta-analysis due to variable formulations and limited transdermal absorption [224].
Metabolic Disorders	Resveratrol, Curcumin, Berberine, Quercetin	Enhance insulin signaling, reduce β-cell apoptosis, inhibit AGEs	T2DM, diabetic neuropathy	Curcumin and resveratrol enhanced pancreatic β-cell function [225]. Berberine lowered FPG and improved insulin sensitivity and glucose metabolism in HFD rats [226].	Curcumin adjunct therapy in RCT improved HbA_1_c by 0.4% over 12 weeks [227].	Meta-analysis: Mild HbA_1_c reduction (<0.5%) from curcumin supplementation with significant heterogeneity [228].
Ocular Health	Lutein, Zeaxanthin, Vit C, Vit E, Zinc, Vit A	Filter blue light, protect RPE, reduce lipid peroxidation	AMD, cataracts	Lutein and zeaxanthin delayed AMD progression and protected photoreceptors in light-exposed RPE cell models [229,230].	RCT: AREDS2 slight reduction in AMD progression from lutein and zeaxanthin [231]. Network meta-analysis supports efficacy in early AMD by improving visual acuity [232].	RCTs: No significant reduction in early AMD with dietary lutein and zeaxanthin [233,234]. Large long-term RCT (AREDS2): No benefit in advanced cataracts from lutein and zeaxanthin [235,236]. RCT (AREDS2), dietary lutein, and zeaxanthin effective only in early/intermediate AMD [236].
Liver Diseases	Silymarin, NAC, Vit E, Glutathione	Restore mitochondrial redox, inhibit lipid peroxidation	NAFLD, NASH, hepatitis	Silymarin reduced hepatic inflammation and fibrosis in NAFLD in CCl_4_-induced injury mouse models [237,238,239].	RCT: Vit E effective treating NASH in adults without diabetes [240]. Meta-analyses: Vit E effective for treatment of NASH and NAFLD in adult and pediatric patients [139,241,242,243].	Large long-term RCT (SELECT): Increased prostate cancer risk with selenium and vit E with modest drop in hepatic inflammation [215].
Reproductive Health	Vit E, CoQ10, Zinc, L-Carnitine	Reduce sperm DNA damage, supports oocyte quality, enhance mitochondrial energetics	Male/female infertility, PCOS	CoQ10 conserved against oxidative stress injury in germinal cells of rats. CoQ10 improved sperm count and motility in rats [244,245].	Meta-analysis: CoQ10 improved sperm motility, concentration, and morphology [246,247].	Meta-analysis: Limited evidence in support of supplemental oral antioxidants for subfertile women; non-significant live birth rate improvements [248]. PCOS studies underpowered due to lack of standardization of methodologies across phenotypes [249]
Muscle Physiology	NAC, Vit C, Vit E, Polyphenols, CoQ10	Scavenge exercise-induced ROS, support mitochondrial biogenesis	Muscle fatigue, f	NAC reduced neuromotor pathology, improved endurance and mitochondrial markers in ethanol and adenine fed mice [250,251].	RCTs: CoQ10 improved physical robustness, balance, gait speed, and symmetry [252,253,254].	High-dose antioxidants blunt training adaptation in RCTs [255,256,257,258,259,260,261]. RCT: CoQ10 ineffective as an ergogenic aid in both young and older trained men [262].
10. Immune Function	Vit C, Vit A, Zinc, Selenium	Regulate redox-sensitive pathways, support leukocyte and NK cell function	Infections, immunosenescence	Vit C enhanced neutrophil activity in stimulated neutrophils and H_2_O_2_-treated HL60 cells [263].	Meta-analysis: Vit C reduced ICU mortality rate in sepsis and ARDS [264,265].	Meta-analyses: No significant benefit on immune function from Vit C in patients with sepsis, viral infections, and common colds [266].
Aging & Frailty	CoQ10, Vit E, Polyphenols	Reduce mitochondrial ROS, preserve telomeres and aging processes	Cognitive decline, frailty, sarcopenia	CoQ10, curcumin, and revesterol extended lifespan and improved cognitive and motor function in aged rodents [267,268,269,270].	RCTs: CoQ10 and selenium reduced serum R-SH levels, reduced cardiac mortality, and improved CV health in elderly [271,272,273,274].	Lifespan extension not seen in human RCTs [207]. Meta-analysis: Minor improvement in physical robustness-related outcomes, but frailty endpoints still exploratory [252].
Inflammatory Diseases	Curcumin, ω-3 FAs, Quercetin, Resveratrol	Inhibit NF-κB, COX-2; ↓ IL-6, TNF-α	RA, IBD, asthma, lupus	Curcumin reduced joint inflammation in mice [275]. Curcumin suppressed TNF-α and IL-6 in DSS-induced colitis and CIA models [276,277].	RCT shows that curcumin decreased serum pro-inflammatory cytokines in subjects with metabolic syndrome [278]. RCT: Meriva^®^ curcumin reduced CRP and DAS28 in RA over 24 weeks [279].	Meta-analysis: Benefit in RA from curcumin but limited in IBD due to low absorption, poor bioavailability, and heterogeneity in RCT endpoints [280].
Renal Disorders	Curcumin, NAC, Vit E, Selenium	Inhibit oxidative glomerular injury	CKD, diabetic mephropathy	Curcumin exhibited renoprotective effects by reducing fibrosis in nephrectomized rats [281,282]	Meta-analysis: Curcumin reduced development of ESKD [283]. RCTs: NAC reduced CKD progression and ESRD in CKD patients [284,285,286].	Meta-analysis: No reduced death or improved kidney transplant outcomes or proteinuria in patients with CKD [283,287]. Curcumin dose adjustment essential in renal impairment. Risk of nephrotoxicity with overdose; hydration status affects outcomes.
Respiratory Disorders	NAC, Quercetin, Vit E, Curcumin	Reduce airway ROS, inhibit cytokine-driven mucus production	COPD, asthma, pulmonary fibrosis	NAC attenuated lung damage, pulmonary emphysema, mucus, and inflammation in COPD rats [288,289,290,291].	Meta-analysis: Inhaled NAC improved FEV_1_ by 7% and reduced exacerbations in moderate COPD [292].	Meta-analysis: NAC did not reduce the risk of acute exacerbation or ameliorate the decline in lung volume in COPD patients [293]. NAC effective only in chronic use, inhaled forms superior to oral. Mixed COPD results with oral NAC; limited bioavailability [294].
Gastrointestinal Health	Curcumin, Sulforaphane, Polyphenols	Preserve gut barrier, modulate microbiota, reduce redox-driven inflammation	IBD, CRC, gastritis	Curcumin maintained intestinal barrier function and effectively alleviated colitis injury in DSS colitis mice [276,295].	RCT: Curcumin + mesalamine induced remission in patients with ulcerative colitis [296].	RCTs are small and heterogeneous; formulation issues common [297].
Skeletal Health	Vit K, Resveratrol, Vit C	Inhibit osteoclast activity, enhance osteoblast mineral deposition	Osteoporosis, inflammatory bone loss	GTP reduced bone loss and improved mineral density and bone mechanical properties in chronic inflammation-induced bone loss mice [298,299].	Meta-analyses: Antioxidants effective for sarcopenia, especially improving muscle strength and function [300,301,302].	RCT: Antioxidant supplementation did not affect bone turnover markers [303,304]; Human data limited; limited FDA-approved antioxidant sfor bone health.

AD = Alzheimer’s disease; PD = Parkinson’s disease; HD = Huntington’s disease; T2DM = type 2 diabetes mellitus; RPE = retinal pigment epithelium; PCOS = polycystic ovary syndrome; IBD = inflammatory bowel disease; CRC = colorectal cancer; LDL = low-density lipoprotein; HFS = high-fat diet; HbA1c = glycated hemoglobin; AMD = age-related macular degeneration; PE = pigment epithelium; NAC = N-acetyl cysteine; NAFLD = nonalcoholic fatty liver disease; NASH = nonalcoholic steatohepatitis; RA = rheumatoid arthritis; CKD = chronic kidney disease; COPD = chronic obstructive pulmonary disease; GTP = green tea polyphenols; FPG = fasting plasma glucose; NF-κB = nuclear factor kappa-light-chain-enhancer of activated B cells; COX-2 = cyclooxygenase-2; IL-6 = interleukin-6; TNF-α = tumor necrosis factor alpha; CIA = collagen-induced arthritis; CRP = C-reactive protein; DAS28 = Disease Activity Score in 28 joints.

## Data Availability

No new data were created.

## Abbreviations

The following abbreviations are used in this manuscript:

GPx	Glutathione peroxidase;
SOD	Superoxide dismutase;
COX-2	Cyclooxygenase-2;
CAT	Catalase;
EGCG	Epigallocatechin-3-gallate;
DNA	Deoxyribonucleic acid;
Nrf2	Nuclear factor erythroid 2-related factor 2;
TNF-α	Tumor necrosis factor-alpha;
IL-6	Interleukin-6;
NF-κB	Nuclear factor kappa B;
GSTs	Glutathione S-transferases;
HO-1	Heme oxygenase-1;
NQO1	NAD(P)H:quinone oxidoreductase 1;
TrxR	Thioredoxin reductase;
SIRT1	Sirtuin 1;
CoQ10	Coenzyme Q10;
GSH	Glutathione;
PON2	Paraoxonase-2;
HIF-1α	Hypoxia-inducible factor 1-alpha;
STAT3	Signal transducer and activator of transcription 3;
FOXO	Forkhead box O;
p53	Tumor protein 53;
IKK	IκB kinase;
AP-1	Activator protein 1;
Keap1	Kelch-like ECH-associated protein 1.
